# Structural and compositional diversity of fibrillin microfibrils in human tissues

**DOI:** 10.1074/jbc.RA117.001483

**Published:** 2018-02-16

**Authors:** Alexander Eckersley, Kieran T. Mellody, Suzanne Pilkington, Christopher E. M. Griffiths, Rachel E. B. Watson, Ronan O'Cualain, Clair Baldock, David Knight, Michael J. Sherratt

**Affiliations:** From the ‡ Division of Cell Matrix Biology and Regenerative Medicine,; the § Division of Musculoskeletal and Dermatological Sciences,; the ‖ School of Biological Sciences, and; the ** Wellcome Trust Centre for Cell-Matrix Research, Faculty of Biology, Medicine and Health, University of Manchester, Manchester M13 9PT, United Kingdom and; the ¶ NIHR Manchester Biomedical Research Centre, Central Manchester University Hospitals NHS Foundation Trust, Manchester Academic Health Science Centre, Manchester M13 9PT, United Kingdom

**Keywords:** extracellular matrix, skin, eye, fibroblast, proteomics, atomic force microscopy (AFM), protein structure, collagen VI, fibrillin microfibril

## Abstract

Elastic fibers comprising fibrillin microfibrils and elastin are present in many tissues, including the skin, lungs, and arteries, where they confer elasticity and resilience. Although fibrillin microfibrils play distinct and tissue-specific functional roles, it is unclear whether their ultrastructure and composition differ between elastin-rich (skin) and elastin-poor (ciliary body and zonule) organs or after *in vitro* synthesis by cultured cells. Here, we used atomic force microscopy, which revealed that the bead morphology of fibrillin microfibrils isolated from the human eye differs from those isolated from the skin. Using newly developed pre-MS preparation methods and LC-MS/MS, we detected tissue-specific regions of the fibrillin-1 primary structure that were differentially susceptible to proteolytic extraction. Comparing tissue- and culture-derived microfibrils, we found that dermis- and dermal fibroblast–derived fibrillin microfibrils differ in both bead morphology and periodicity and also exhibit regional differences in fibrillin-1 proteolytic susceptibility. In contrast, collagen VI microfibrils from the same dermal or fibroblast samples were invariant in ultrastructure (periodicity) and protease susceptibility. Finally, we observed that skin- and eye-derived microfibril suspensions were enriched in elastic fiber– and basement membrane–associated proteins, respectively. LC-MS/MS also identified proteins (such as calreticulin and protein-disulfide isomerase) that are potentially fundamental to fibrillin microfibril biology, regardless of their tissue source. Fibrillin microfibrils synthesized in cell culture lacked some of these key proteins (MFAP2 and -4 and fibrillin-2). These results showcase the structural diversity of these key extracellular matrix assemblies, which may relate to their distinct roles in the tissues where they reside.

## Introduction

Extracellular matrices (ECM)^3^ are commonly composed of a diverse array of assemblies, which make key contributions to tissue mechanics and cell-mediated homeostasis. Some of these assemblies, such as the fibrillar collagens and the elastic fibers, are large, insoluble, and supramacromolecular. Some are markedly long-lived, laid down early in development, where they persist and undergo a process of maturation (1) and subsequent age- and disease-related accumulation of damage (2, 3). During these processes, the ultrastructure of these assemblies can be tissue-specific (1). Therefore, although these ECM assemblies are present in multiple tissues, they may exhibit distinct development-mediated ultrastructures that have evolved to fulfill their unique functionality.

Elastic fibers (composed of fibrillin microfibrils and elastin (4)) are present in many tissues, including skin (5), lungs (6), arteries (7), and ligaments (8), where they play a major role in conferring elasticity and resilience (4). The fibrillin microfibril, along with elastin, is a key component of the elastic fiber and adopts a bead-on-a-string appearance (9) when extracted and viewed with atomic force microscopy (AFM) and EM. Additionally, these microfibrils exist also as stand-alone assemblies, forming candelabra-like structures (10) (for a review, see Ref. 11) in the papillary dermis. They also play a role in tissue homeostasis, sequestering and storing the latent forms of members of the TGF-β (12, 13) and BMP families (14). In eyes, fibrillin microfibrils play an architectural role very different from that in skin. They form the ciliary zonules, stand-alone suspensory ligaments that connect the lens capsule to the ciliary muscle (15). These zonules come under tensile stress as the ciliary muscle exerts a strain to deform the lens during accommodation. Although fibrillin microfibrils appear structurally and compositionally similar in mammalian tissues and cell culture systems and retain a beadlike structure (and presence of the main component, fibrillin-1) throughout different tissues (9), little is known about whether they have evolved to be distinct in each. Only two studies have shown that fibrillin microfibril ultrastructure is tissue- and developmentally dependent. In 1997, we showed that intertissue differences in mass and periodicity (interbead distance) exist in microfibrils derived from bovine fetal aorta and skin (16). We also showed that fibrillin microfibrils undergo a process of post-translational maturation as their mass increases during fetus development. Lu *et al.* (17) also reported similar differences in bead morphology between bovine adult aorta- and ciliary zonule-derived fibrillin microfibrils.

Because fibrillin microfibrils are present in a variety of tissues, the different roles they play may be reflected in the ultrastructure they adopt. These intertissue comparisons have never been made in humans or between fibrillin microfibrils sourced from ciliary body (CB) and skin, where they play very different architectural and mechanical roles. Additionally, the fibrillin microfibril's biomolecular composition has never been compared between tissues. Although their ultrastructure has been extensively studied using AFM (18–20) and EM (9, 18, 21), characterization of the biomolecular composition through conventional biochemical approaches such as gel electrophoresis can be problematic due to their large size and insolubility. As a consequence, it is necessary to develop proteomic approaches to characterize fibrillin microfibril composition.

Recently, De Maria *et al.* (22) performed whole-tissue proteomics on dissected human and bovine ciliary zonules and effectively characterized the zonular proteome. However, to date, only a single published proteomic study, performed by Cain *et al.* (23), has attempted to characterize both the structure and composition of fibrillin microfibrils purified from human tissue. Through LC-MS/MS, Cain *et al.* (23) achieved a 30% primary coverage of fibrillin-1 and identified several microfibril-associated proteins, such as microfibril-associated protein 2 (MFAP2). They demonstrated that MS-based proteomic approaches have the potential to identify the proteins involved in these supramolecular ECM assemblies. However, they observed that peptide generation from the core fibrillin-1 proteins, and their interacting proteins, was challenging due to their large size and high number of cross-links (24). Since this study took place, over 10 years ago, advances have been made in mass spectrometer technology allowing greater resolving power with expanded functionalities (25). Coupled with improved sample preparation, we believe that these proteomic approaches can be enhanced further to allow effective intertissue comparisons of fibrillin microfibril composition and structure.

In this study, we optimized two effective methods of pre-MS sample preparation: elastase digestion and SMART^TM^ digestion, for the enhanced generation of fibrillin peptides and their microfibril-associated proteins. This led to an improved compositional analysis via LC-MS/MS, compared with Cain *et al.* (23). We go on to test differences between the ultrastructure (bead morphology and interbead periodicity) using AFM and biomolecular composition (fibrillin-1 structural and enzymatic susceptibly and associated protein presence) using MS of fibrillin microfibrils isolated from human eye (CB), human skin, and cultured human dermal fibroblasts (HDFs). Because collagen VI microfibrils co-purify with fibrillin microfibrils in skin- and HDF-derived samples, we use them as a comparative control. We perform these analyses to test the following hypotheses: 1) fibrillin microfibril ultrastructure and composition are tissue-dependent and 2) culture-derived, newly synthesized fibrillin microfibril ultrastructure and composition are distinct from those of native, mature, tissue-sourced microfibrils.

## Results and discussion

### Elastase digestion methods enhance fibrillin-1 peptide generation and, combined with SMART^TM^ digestion methods, enhance the detection of microfibril-associated proteins

**Figure 1.**
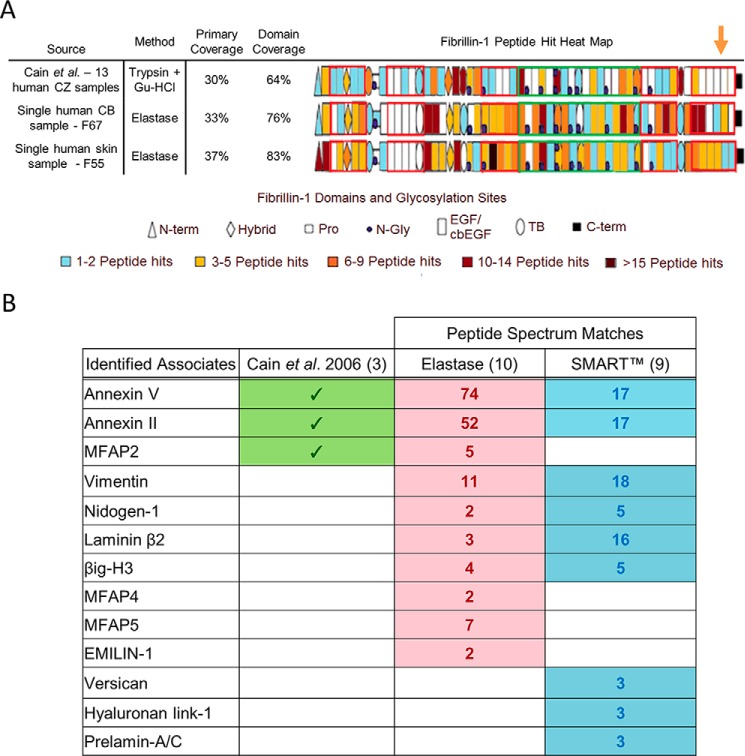
**Elastase and SMART^TM^ methods led to the improved detection of fibrillin-1 and improved identification of microfibril-associated proteins compared with previous published efforts.** The ability of the elastase method to produce peptides of fibrillin-1 from a single human CB sample (female age 67; F67) and a single human skin sample (F55) is compared with the efforts of Cain *et al.* (23) (*A*). As performed by Cain *et al.* (23), PSMs (Peptide Prophet FDR ≤ 5%) were counted for each respective fibrillin-1 domain and heat-mapped. Our method led to a greater primary coverage and domain coverage from a single sample run than Cain *et al.* (23), whose coverages were achieved from 13 separate sample runs. Peptides from the C-terminal region of fibrillin-1 were also successfully detected (*orange arrow*), which Cain *et al.* (23) failed to identify. The known fibrillin microfibril-interacting proteins identified by Cain *et al.* (23) (*green*) are compared with those identified by elastase and SMART^TM^ methods (Protein Prophet FDR ≤ 0.1%, Peptide Prophet FDR ≤ 5%) (*B*). The elastase method (*red*) and SMART^TM^ method (*blue*) were both performed on the same human CB microfibril extract (F73). The elastase method appears to enhance the detection of microfibril-associated proteins thought to be tightly bonded to the structure (*i.e.* the MFAP family), whereas the SMART^TM^ method appears to enhance the detection of weakly interacting proteins (*i.e.* versican and hyaluronan proteins). Collectively, these methods led to an enhanced detection of known microfibril-associated proteins compared with Cain *et al.* (23).

To improve the generation of peptides from core microfibril components, porcine elastase, a highly active and nonspecific enzyme (which preferentially cleaves leucine, isoleucine, alanine, serine, valine, and glycine) (26, 27) was used instead of conventional trypsin-based methodologies (for a review, see Ref. 28). For human CB-derived fibrillin microfibrils, this method, along with the use of a latest-generation mass spectrometer, led to an improved primary coverage (33%) and domain coverage (76%) of fibrillin-1, compared with that achieved by Cain *et al.* (23) (30% primary, 64% domain) (Fig. 1*A*) and, for the first time, the identification of peptides from the C-terminal region of fibrillin-1 (*orange arrow*). A similar primary sequence and domain coverage were achieved when applied to human skin. Crucially, this improved coverage was achieved by the digestion and MS of single CB and skin samples, whereas Cain *et al.* (23) reported a total primary sequence coverage of 30% from peptides identified in 13 separately prepared human Gu-HCl– and/or trypsin-treated ciliary zonule samples.

To improve peptide generation from proteins that co-purify with the microfibril, SMART^TM^ digestion was used. Collectively, the elastase and SMART^TM^ digestion methods led to the successful identification of 13 known microfibril-associated proteins (Fig. 1*B*) from human CB. These include annexin V, annexin II, and MFAP2, identified by Cain *et al.* (23) in 2006.

### Fibrillin microfibril bead morphology is tissue-dependent

**Figure 2.**
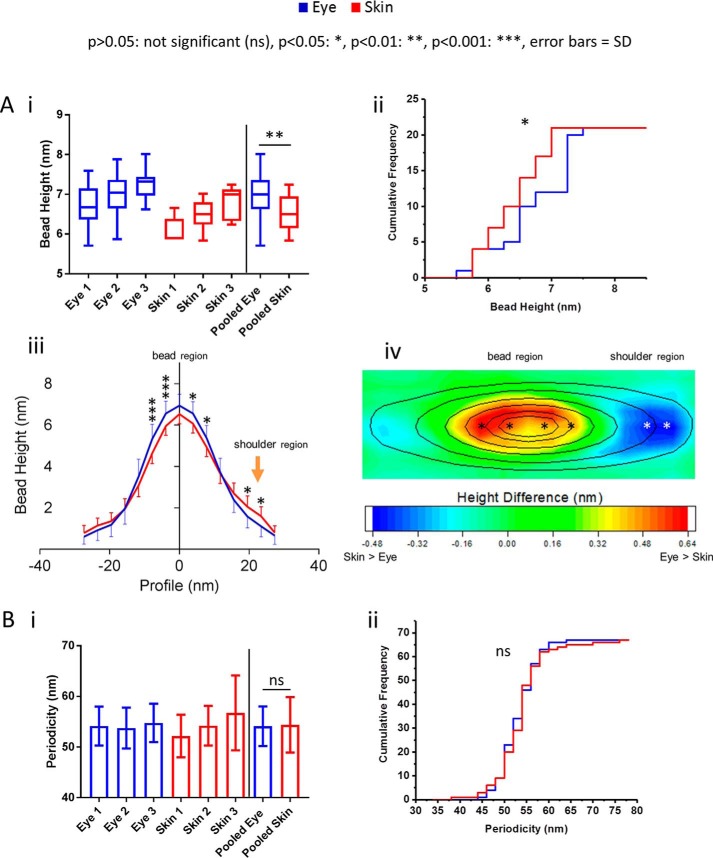
**Fibrillin microfibril ultrastructure is tissue source–dependent.** Eye-derived fibrillin microfibril mean central bead height was significantly higher (6.94 nm; *n* = 100 repeats/sample, averaged per microfibril; *n* = 300 repeats in pooled data) than skin-derived (6.51 nm; *p* = 0.0023, Mann–Whitney *U* test) (*A*, *i*). Cumulative frequency distributions of central bead height (averaged per microfibril, *n* = 21 pooled) indicate a large population of eye-derived fibrillin microfibrils with significantly larger beads (*p* = 0.0423; Kolmogorov–Smirnov) than skin-derived (*A*, *ii*). Axial bead profiles show that, although skin-derived fibrillin microfibril beads are significantly smaller, within ∼10 nm of the bead peak, than eye-derived (*A*, *iii*; Bonferroni corrected multiple comparison test), they also have significantly higher slopes at the shoulder regions than eye-derived (*orange arrow*). This may suggest that skin-derived fibrillin microfibril beads have a different volume distribution than that of eye-derived. To visualize these differences in bead morphology, the whole AFM height maps of skin-derived fibrillin microfibril beads were averaged and subtracted from that of eye-derived. The resulting height differences are represented as a heat map overlaid with the average height contour of the eye-derived beads (*A*, *iv*). The significant differences seen in the axial profile panel (*iii*) were also added to the heat map (*iv*, *stars*). The biggest differences in bead morphology were around the central peak, where eye-derived beads were higher than skin-derived, and at the shoulder region, where skin-derived beads were higher than eye-derived. There was no significant difference (*ns*) in the mean periodicity (*p* = 0.9737; Mann–Whitney *U* test, *n* = 500 repeats/sample, averaged per microfibril; *n* = 1500 repeats in pooled data) (*B*, *i*) and in the cumulative frequency distributions of periodicities (*p* = 0.8580; Kolmogorov–Smirnov, averaged per microfibril, *n* = 67 pooled) (*B*, *ii*) between eye- and skin-derived fibrillin microfibrils. *Error bars*, S.D.

The fibrillin microfibril is composed predominantly of fibrillin-1 (∼8 monomers per single bead and interbead repeat) with a total mass of ∼2.5 MDa per repeat (9). The average periodicity and bead width has been approximated to 56 and 19 nm, respectively (for a detailed breakdown of microfibril dimensions, see Ref. 9). Our data showed that fibrillin microfibrils derived from human eye (CB) had a significantly higher mean central bead height than those derived from human skin. (Fig 2*A*, *i*). Central bead height frequency distributions indicate that the majority of eye-derived fibrillin microfibrils had larger beads than skin-derived (Fig. 2*A*, *ii*). Additionally, average axial height profiles showed that although eye-derived fibrillin microfibrils beads are significantly higher within a ∼10-nm radius of the center, they were significantly lower at the shoulder region, ∼20 nm from the peak (Fig. 2*A*, *iii*, *orange arrow*) than skin-derived. These height differences in bead morphology are further exemplified in the contour heat map (Fig. 2*A*, *iv*), where eye-derived microfibrils appear to have a more pronounced bead with a lower shoulder region than skin. Between beads, however, there was no significant difference in the mean periodicity of fibrillin microfibrils derived from eye and skin (Fig. 2*B*, *i* and *ii*)

Many past studies exclusively used differences in periodicity to gauge ultrastructural differences in fibrillin microfibrils (19, 29–32) and other fibrillar components of the ECM (19, 33–35). However, not only does the majority of the fibrillin microfibril's mass rest within the bead, much of microfibril's functionality is thought to be mediated via the interaction between the bead and its associated proteins (36–38). Our data showed that eye-derived fibrillin microfibril beads differ in morphology in comparison with skin-derived, but periodicity did not. By omitting analysis of the microfibril bead, these studies may have missed some key ultrastructural changes linked to health and disease.

The ultrastructural variances seen between the beads of adult human eye- and skin-derived fibrillin microfibrils are similar to those we observed previously (16), where differences were detected in bead mass of microfibrils from bovine fetal skin and aorta. Lu *et al.* (17) also detected differences in bead morphology, including the shoulder regions, from bovine adult ciliary zonule and aorta.

### Fibrillin-1 derived from human eye and skin exhibit intertissue, regional differences in elastase susceptibility

To further compare and substantiate the ultrastructural differences seen between CB and skin fibrillin microfibrils, it was necessary to characterize at their biomolecular composition (fibrillin-1 structure and known microfibril-associated protein presence). Previous studies have used differences in fibrillin-1's susceptibility to proteolysis to gauge abnormalities in fibrillin microfibril structure (39) and function (40, 41). It is possible that the fibrillin-1 structure may exhibit regional differences in proteolytic susceptibilities depending on its tissue of origin.

**Figure 3.**
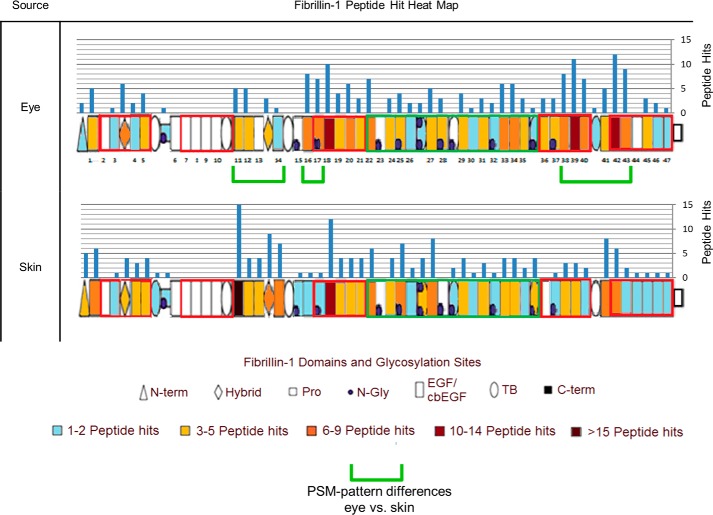
**Eye-derived fibrillin-1 exhibits different regional patterns of elastase susceptibility compared with skin-derived.** LC-MS/MS–detected fibrillin-1 PSMs (Peptide Prophet FDR ≤ 5%) were counted for each respective protein domain, per sample (*n* = 3), averaged (normalized based on total spectrum count), and subsequently heat-mapped to their corresponding domain. Eye-derived fibrillin-1 yielded more peptides between epidermal growth factor-like domains 38 (EGF38) and EGF43 than skin-derived (53 total from eye-derived *versus* 24 total from skin-derived) (*green brackets*). Eye-derived fibrillin-1 also yielded more peptides at EGF16 and EGF17 than skin-derived (15 *versus* 2); however, it yielded fewer peptides between EGF11 and EGF14 (14 *versus* 39).

LC-MS/MS–detected peptide hit patterns (23) indicate several regions of human eye-derived fibrillin-1 (Fig. 3) with differing susceptibilities to elastase in comparison with skin-derived (*green brackets*). These regional differences indicate that, not only are fibrillin microfibrils ultrastructurally variable between tissues, but their fibrillin-1 structure may also be as well. Collectively, the differences in structure suggest that these supramolecular assemblies may have evolved distinct ultrastructures and compositions to cope with their different architectural, mechanical, and biochemical roles in their respective tissues of origin. It is possible that the presence of different cell types within each tissue may have contributed to these differences. Baldwin *et al.* showed that the epithelial-mesenchymal state of retinal pigment epithelial cells influenced their ability to assemble fibrillin microfibrils (42). Although fibroblasts (mesenchymal cells) are thought to be responsible for microfibril deposition in skin (43) and in eye (44), it remains unknown whether epithelial cells contribute to fibrillin microfibril synthesis *in vivo* (45). It is also unclear whether populations of fibroblasts from different tissues exhibit differences in epithelial-mesenchymal states, as shown in retinal pigment epithelial cells (42). It is possible, therefore, that different cell types (or cells in different states) may synthesize fibrillin microfibrils with localized differences in structure.

So far, we have demonstrated that fibrillin microfibril ultrastructure and fibrillin-1 regional susceptibility are tissue-dependent. These differences may also relate to microfibril post-translational maturation in development. To study this, we applied the same analysis to newly synthesized fibrillin microfibrils derived from cultured HDFs and compared them with skin fibrillin microfibrils, derived *ex vivo*.

### Newly synthesized, HDF-derived fibrillin microfibrils exhibit marked differences in ultrastructure compared with skin-derived

**Figure 4.**
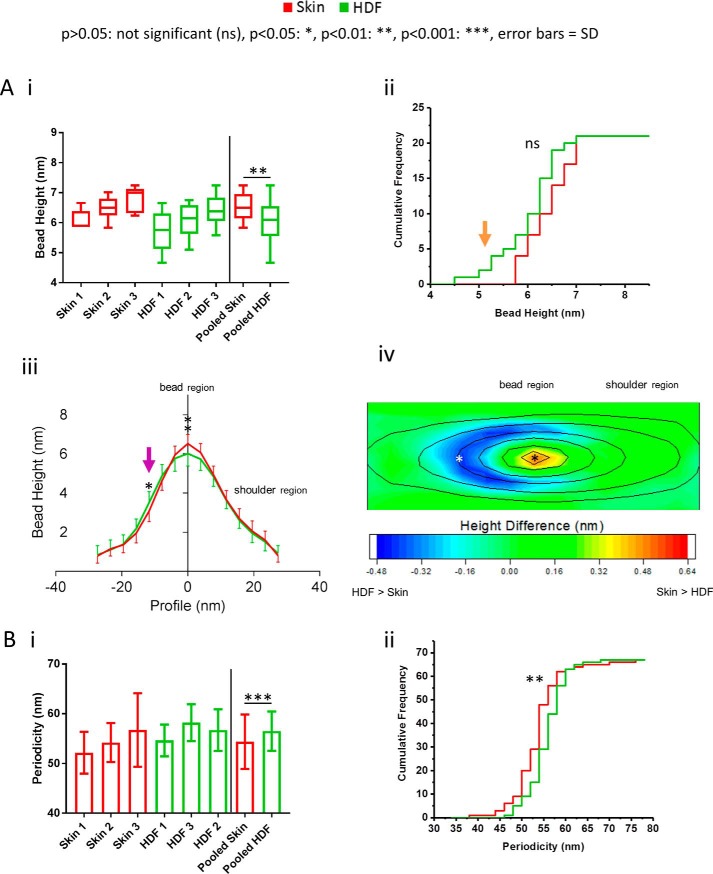
**Newly synthesized, HDF-derived fibrillin microfibril ultrastructure is significantly different from native skin-derived.** HDF-derived fibrillin microfibrils had significantly lower central bead heights (6.02 nm; *n* = 100 repeats/sample, averaged per microfibril; *n* = 300 repeats in pooled data) than skin-derived (6.51 nm, *p* = 0.0038, Mann–Whitney *U* test) (*A*, *i*). Cumulative frequency distributions of central bead height were not significantly different (*p* = 0.1938, Kolmogorov–Smirnov; averaged per microfibril, *n* = 21 pooled); however, they indicate a subpopulation of HDF-derived fibrillin microfibrils with smaller beads than skin-derived (*A*, *ii*; *orange arrow*). Axial bead profiles (*A*, *iii*) show that skin-derived fibrillin microfibril beads are significantly higher than HDF-derived beads close to the central peak and significantly lower than HDF-derived beads at the slope opposite to the shoulder region (*purple arrow*) (Bonferroni corrected multiple-comparison test; *n* = 300 repeats pooled, averaged per microfibril). These changes in bead morphology are reflected in the height difference contoured heat map (*A*, *iv*) (AFM height maps of HDF-derived fibrillin microfibril beads were averaged and subtracted from that of skin-derived and subsequently heat-mapped; the contour height of the average skin bead was then overlaid). Skin beads were higher than HDF beads only near the central peaks, whereas HDF beads had higher slopes around the peaks (except near the shoulder region). HDF-derived fibrillin microfibrils exhibited a significantly higher periodicity (56.5 nm; *n* = 500 repeats/sample, averaged per microfibril; *n* = 1500 repeats in pooled data) compared with skin-derived (54.4 nm; *p* = 0.0004, Mann–Whitney *U* test) (*B*, *i*). Periodicity cumulative frequency distributions indicate a large population of HDF-derived fibrillin microfibril with significantly higher periodicities (*B*, *ii*) in comparison with skin-derived (*p* = 0.0051; Kolmogorov–Smirnov, averaged per microfibril, *n* = 67 pooled). *Error bars*, S.D.

On average, cultured HDF-derived, newly synthesized, fibrillin microfibrils had a significantly lower central bead height than human skin-derived (Fig. 4*A*, *i*). Additionally, central bead height frequency distributions indicate a subpopulation of cultured HDF-derived fibrillin microfibrils with smaller beads than human skin-derived (Fig. 4*A*, *ii*, *orange arrow*). Average axial height profiles indicate that, although skin fibrillin microfibril beads have a significantly larger central peak height than cultured HDF-derived microfibrils, the reverse is true on the slopes of the beads (opposite to the shoulder region) (Fig. 4*A*, *iii*, *purple arrow*). This difference in bead morphology is further shown in the contour heat map (Fig. 4*A*, *iv*). Skin-derived beads have a higher peak with a more pronounced slope (except near the shoulder region) than HDF-derived beads. This indicates that beads of newly synthesized fibrillin microfibrils from cultured-HDFs have a morphology different from those derived from human skin.

On average, cultured HDF-derived fibrillin microfibrils also exhibited a significantly higher periodicity in comparison with skin-derived fibrillin microfibrils (Fig. 4*B*, *i*). In addition, periodicity frequency distributions show a large population of cultured HDF-derived fibrillin microfibrils with significantly higher periodicities than skin-derived (Fig. 4*B*, *ii*).

Similar differences in fibrillin microfibril bead morphology and periodicity have been reported previously in three cases. The first is between tissues where we showed that bovine fetal aorta fibrillin microfibrils had a higher bead mass and a lower periodicity than those derived from skin (16) and where Lu *et al.* (17) also reported that aorta-derived fibrillin microfibrils had differing bead morphologies and a higher periodicity compared with those from bovine zonules. The second is during developmental microfibril maturation, where we also showed that the gradual increase in fetal fibrillin microfibril bead mass and the gradual decrease in periodicity correlated with gestation time (16). The third is during photoaging, where two studies highlighted the structural susceptibility of fibrillin microfibrils to UV irradiation. We showed that a low-dose UVB irradiation of both HDF- and human skin-derived fibrillin microfibrils directly led to the marked loss and redistribution of their bead mass and a significant increase in their periodicity (20). Since then, our group has also showed that physiological doses of both solar-simulated radiation (∼5% UVB and ∼95% UVA) and pure UVA led to a significant decrease in the periodicity of HDF-derived fibrillin microfibrils (19).

Although these fibrillin microfibril ultrastructural differences have been reported between tissues, during maturation, and in photodamage, this study has identified them between microfibrils derived *in vitro*, from primary fibroblasts (natively found in human skin), and those derived *ex vivo*, directly from human skin. It is possible, therefore, that 1) the fibrillin microfibrils generated by HDFs are structurally immature in comparison with native microfibrils sourced from skin (either through lack of development or through the cell culture process) or that 2) the native skin-derived microfibrils have accumulated structural damage during aging in comparison with those newly synthesized from HDFs. The aging process would be more intrinsic than extrinsic (photoaging) because abdominal skin is relatively photoprotected compared with forearm skin used in previous photoaging studies (5). Because elastic fiber production is commonly thought to be fibroblast-driven (43), these changes may have profound implications for skin-regenerative therapies, especially if they are linked to developmental maturation or aging.

### Fibrillin-1 derived from newly synthesized, HDF fibrillin microfibrils exhibited regional differences in elastase susceptibility compared with skin-derived

**Figure 5.**
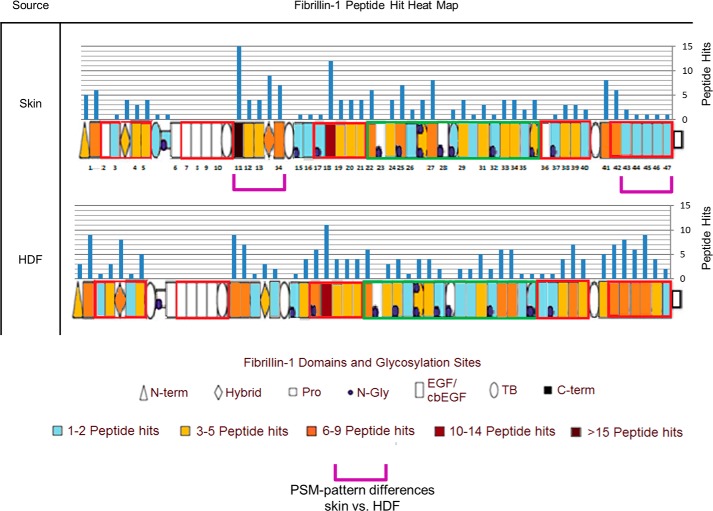
**Fibrillin-1, derived from newly synthesized HDF fibrillin microfibrils, exhibits different regional patterns of elastase susceptibility compared with skin-derived.** LC-MS/MS–detected fibrillin-1 PSMs (Peptide Prophet FDR ≤ 5%) were counted for each respective protein domain, per sample (*n* = 3), averaged (normalized based on total spectrum count), and subsequently heat-mapped to their corresponding domain. Skin-derived fibrillin-1 yielded fewer peptides from the last five domains at the C-terminal region, between EGF43 and EGF47 (6 *versus* 29), than HDF-derived; however, skin-derived yielded more peptides between EGF11 and EGF14 (39 *versus* 22) than HDF-derived (*purple brackets*).

LC-MS/MS–detected peptide hit patterns (23) indicate several regions of human skin-derived fibrillin-1 (Fig. 5) with differing susceptibilities to elastase in comparison with HDF-derived (*purple brackets*). The observations that 1) these regions of cultured HDF-derived fibrillin-1 have a structural susceptibility to elastase different from skin-derived and 2) HDF-derived fibrillin microfibrils have different ultrastructures compared with skin (Fig. 4) both support the possibility that either newly synthesized fibrillin microfibrils, derived from HDFs, are structurally immature compared with the more developmental (46), long-lived (2) microfibrils from skin or that the skin-derived fibrillin microfibrils are exhibiting signs of aging in comparison with those newly synthesized from cells.

It is possible that these long-lived (2), skin-derived fibrillin microfibrils may have accumulated age-related damage through the formation of oxidative cross-links (47) (for a review, see Ref. 48) induced by the long-term exposure to reactive oxygen species in tissue and also via the accrual of advanced-glycation end products on fibrillin-1 (49) (for a review, see Ref. 50). This process may have led to a differential susceptibility to enzyme digestion and to an accrual of sugar on the surface of the bead, which would explain the variations in bead morphology.

### HDF and skin-derived collagen VI microfibril structure is conserved compared with the fibrillin microfibril

Like the fibrillin microfibril, tissue collagen VI microfibrils are long-lived (3), supramolecular, beaded assemblies (52). Both microfibrillar species are highly abundant in connective tissue (53) and, as such, regularly co-purify (19, 53). This allowed us to a make a useful comparison between periodicity differences in collagen VI microfibrils and periodicity differences of fibrillin microfibrils in the same skin- and HDF-derived samples. However, because collagen VI microfibril beads are relatively small in comparison with fibrillin microfibril beads (19), unfortunately, AFM resolution was not good enough to assess differences in collagen VI bead morphology. Encouragingly, however, the optimized elastase method generated sufficient collagen VI α-3 (COL6A3) peptides (Fig. S1) to enable its regional susceptibility to elastase to also be compared with fibrillin-1. These comparisons allow us to differentiate whether the changes seen so far, between newly synthesized fibrillin microfibrils in culture and those derived in tissue, extrapolate to another predominating component of the ECM.

**Figure 6.**
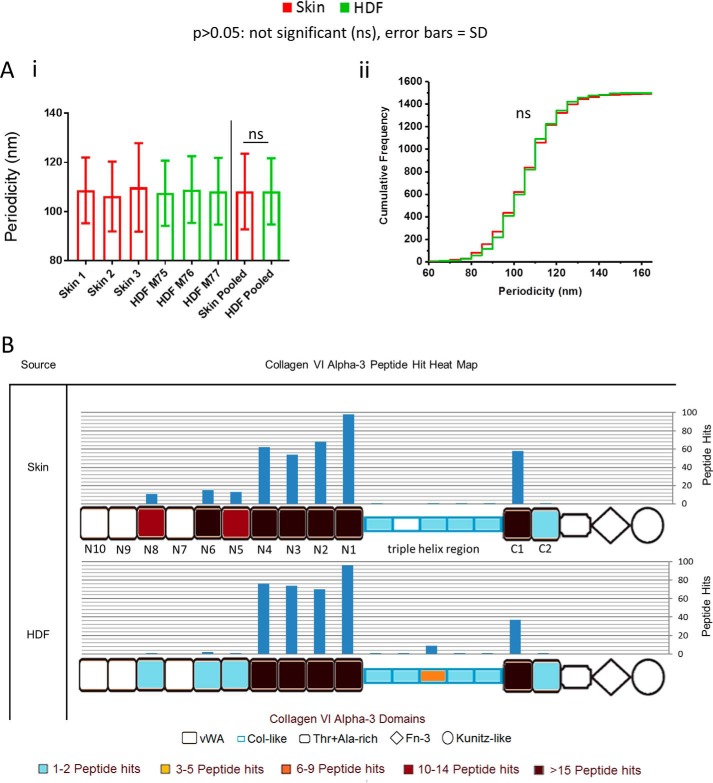
**HDF- and skin-derived collagen VI microfibril ultrastructure and its susceptibility to elastase is predominantly invariant.** There was no significant difference (*ns*) in periodicity between HDF-derived (108.2 nm; *n* = 500 repeats/sample, averaged per repeat; *n* = 1500 repeats in pooled data) and skin-derived (108.2 nm; *p* = 0.6310, Mann-Whitney *U* test) collagen VI microfibrils (*A*, *i*). Additionally, there was no significant difference between periodicity-cumulative frequency distributions of HDF- and skin-derived collagen VI microfibrils (*p* = 0.2656, Kolmogorov–Smirnov) (*A*, *ii*). LC-MS/MS–detected collagen VI α-3 (COL6A3) peptide sequences (Peptide Prophet FDR ≤ 5%) were counted for each respective protein domain, per sample (*n* = 3), averaged (normalized based on total spectrum count), and subsequently heat-mapped to their corresponding domain (*B*). There were similar PSM patterns between skin- and HDF-derived COL6A3 in all regions except at domains N5, N6, and N8, which yielded more peptides from skin-derived (39 total) than HDF-derived (four total). This analysis could not be effectively performed on eye-derived samples, due to low abundance of collagen VI microfibrils.

The periodicity and elastase susceptibility differences seen between HDF- and skin-derived fibrillin microfibrils (Fig. 4*B*) are in stark contrast to the lack of differences seen in collagen VI microfibrils within the same samples (Fig. 6*A*). Newly synthesized, cultured HDF-derived collagen VI microfibril periodicity was not significantly different from that of skin collagen VI microfibrils, derived *ex vivo* (Fig. 6*A*, *i*). In addition, no distinctly different subpopulations of collagen VI microfibrils were seen when looking at periodicity frequency distributions of cultured HDF- and skin-derived collagen VI microfibrils (Fig. 6*A*, *ii*). In fact, both distributions follow almost the same pattern, suggesting that there is very little difference in the periodicity of collagen VI microfibrils from these two sources, unlike the fibrillin microfibril (Fig. 4*B*).

LC-MS/MS–detected peptide hit patterns, in response to elastase digestion of COL6A3 (Fig. 6*B*), were similar at the triple-helix region (52), at the N1–N4 region, and at the C1 domain of both skin- and HDF-derived samples. This is, again, in contrast to fibrillin-1, which did exhibit regional differences in response to elastase digestion (Fig. 5). However, domains N5, N6, and N8 yielded many more peptides from skin-derived COL6A3 than from cultured HDF-derived (Fig. 6*B*). Alternative splicing of COLA3 has been shown previously both in mice and in humans (54), and isoforms of this COL6A3 lacking domains N5 and N7–N10 have been identified previously in human cell lines (54, 55). It is possible, therefore, that cultured HDFs are also synthesizing collagen VI microfibrils that are lacking these domains, which would explain the reduction in peptide hits seen from these regions in the HDF-derived preparations compared with skin.

Previously, we have shown that collagen VI microfibril ultrastructure (periodicity) is resistant to both UVA and solar-simulated radiation, whereas fibrillin microfibril ultrastructure is susceptible (19). Additionally, Watson *et al.* (56) demonstrated that collagen VI microfibril distribution is unaffected in photoaged skin, also in contrast to fibrillin microfibrils in elastic fibers, which are markedly reduced in photoaged skin (5). Kielty *et al.* (57) also reported that skin-derived collagen VI microfibril ultrastructure was indistinguishable at each stage of bovine fetal development, unlike fibrillin microfibril ultrastructure, which is (16). The observations made in this study, that newly synthesized, HDF-derived collagen VI microfibrils are structurally similar to the long-lived microfibrils derived from tissue, corroborate evidence that they are resistant to age-related damage accumulation and that their ultrastructure may not undergo the developmental process of maturation seen in other components of the ECM. These findings are divergent in comparison with the degradation seen in fibrillin microfibrils in aging and to their maturation process (16) and accentuate the complexity of the fibrillin microfibril in tissue development and aging.

### Differences in the presence of co-purifying microfibril-associated proteins may provide insight into tissue functions of fibrillin microfibrils

So far, differences have been observed in both the ultrastructure of fibrillin microfibrils and their fibrillin-1 regional susceptibility to elastase, derived, *ex vivo*, from eye and skin and *in vitro* from cultured HDFs. The fibrillin microfibril's function is tied to the network it forms with a wide variety of proteins within the ECM (see the references cited in Table 1). It is possible, therefore, that gauging the presence of these associated proteins may provide insight into the role they play within a specific tissue and into the underlying composition of the fibrillin microfibril.

**Table 1:** **Published fibrillin microfibril-associated proteins identified in eye, skin, and HDF microfibril samples** Proteins, detected using LC-MS/MS (Protein Prophet FDR ≤0.1%), along with their peptide hit score (sum of *n* = 3) are shown. Peptide hit scores are as follows: *, 2–5; **, 6–14; ***, >15.

Protein	Published interaction	Microfibril-associated protein presence		
		Eye	Skin	HDF
Annexin A2	Cain *et al.* (58)	***	***	***
Annexin A5	Cain *et al.* (58)	***	***	***
Vimentin	Cain *et al.* (58)	***	***	***
Protein-disulfide isomerase	Meirelles *et al.* 2016 (59)	***	***	***
Calreticulin	Ashworth *et al.* (60)	***	***	***
MFAP5 (MAGP2)	Penner *et al*. (61)	**	***	***
βig-h3	Cain *et al.* (58)	*	***	***
Versican	Isogai *et al.* (37)	*	***	***
MMP14	Ashworth *et al.* (30)	*	**	***
Prelamin-A/C	Cain *et al.* (58)	*	*	**
Vitronectin	Dahlbäck *et al.* (62)	***	**	
MFAP2 (MAGP1)	Trask *et al.* (63)	**	**	
MFAP4	Pilecki *et al.* (64)	*	***	
Fibrillin-2	Zhang *et al.* (46)	*	**	
Laminin β2	Tiedemann *et al.* (65)	***		*
SERBP1	Cain *et al.* (58)	*		*
IGFBP7	Cain *et al.* (58)	*		*
Fibulin-2	Reinhardt *et al.* (66)		**	**
Laminin α5	Tiedemann *et al.* (65)	***		
Nidogen-1	Tiedemann *et al.* (65)	**		
Perlecan	Tiedemann *et al.* (65)	**		
Hyaluronan link protein 1	Ohno-Jinno *et al.* (67)	*		
LTBP2	Hirani *et al.* (68)	*		
Elastin	Sakai *et al.* (21)		*	
Fibulin-1	Roark *et al.* (69)		*	
EMILIN-2	Schiavinato *et al.* (71)		*	
Fibronectin 1	Sabatier *et al.* (70)			***
Thrombospondin 1	Cain *et al.* (58)			***
MMP2	Ashworth *et al.* (30)			**
MMP3	Ashworth *et al.* (30)			*
Decorin	Trask *et al.* (63)			*

Within the eye, skin, and HDF microfibril purifications, a large variety of known fibrillin microfibril-associated proteins (21, 30, 37, 46, 58–71) were identified using LC-MS/MS (Table 1). A large proportion of these associated proteins were uniquely detected in either tissue. Four proteins key to elastic fiber biology were identified in skin: the elastic fiber component elastin (21); elastin microfibril interface-located protein (EMILIN)-2, key to the microfibril's deposition onto elastic fibers (71); fibulin-1 (69), which exists as an interface between elastin and the fibrillin microfibril; and fibulin-2 (66), which co-localizes with elastic fibers *in vivo.* This indicates that fibrillin microfibrils play a dominant role as an elastic fiber component in skin. Conversely, four basement membrane proteins were identified in eye-microfibril samples: perlecan, which was shown to connect fibrillin microfibrils directly to basal laminas, along with two laminins and nidogen-1 which bind to perlecan itself (65). This indicates that fibrillin microfibrils play a major role in linking basement membranes within the CB epithelium of the eye.

The advantage of size-exclusion chromatography–purified microfibril proteomic analysis over whole tissue is that we can state with high confidence that the associated proteins identified must have been bound to the fibrillin microfibrils. Many of the proteins (fibrillin-2, MFAP2, MFAP5, and LTBP2) that directly co-purified with eye-derived microfibrils (Table 1) were the same as those found in the human zonule proteome published by De Maria *et al.* (22). Two of these proteins, metalloproteinase inhibitor 2 (TIMP3) and α-2 macroglobulin (A2M) were also identified in these suspensions (Table S1); however, they had no previously published interactions with fibrillin microfibrils. Because these proteins (TIMP3 in particular) were two of the most abundant protease inhibitors found in their whole-zonule proteome, they could be newly identified associated proteins of the fibrillin microfibrils. However, some of most abundant glycoproteins identified by De Maria *et al.* (22) (emilin-1 and hemicentin-1) in their zonule proteome did not co-purify with our eye-derived fibrillin microfibrils. It could be that these proteins do not associate with the microfibrils directly or that the enzymatic extraction process and purification procedures stripped them from the microfibrils.

Many of the detected fibrillin microfibril-associated proteins were shared between tissues (Table 1). This may provide a key insight into identifying the integral components, fundamental to fibrillin microfibril assembly and function, regardless of the tissue of origin. Fibrillin microfibril assembly begins with the secretion of the fibrillin-1 monomer from the cell (60, 72), where it is N- and C-terminally processed by furin. It is proposed that the fibrillin-1 monomers then dimerize in the extracellular space (60) and that these dimers then form the basic intermediates for further microfibril assembly. This process is thought to occur at the cell surface through the homotypic interaction between the N and C termini of fibrillin-1 dimers (24). Microfibril assembly is thought to be cell-driven, as previous studies have shown that the deposition of fibrillin microfibrils by fibroblasts requires both fibronectin and the RGD-dependent α5β1 integrins (70). The molecular chaperone calreticulin and the disulfide bond–forming protein-disulfide isomerase (PDI), (60) were identified in skin, eye, and HDF microfibril samples. Ashworth *et al.* (60) showed that both of these intracellular proteins bind to fibrillin-1, thereby inhibiting their dimerization and preventing their intracellular aggregation. They go on to propose that when the fibrillin-1 monomers are then secreted from the cell, the loss of these binding partners leads to their dimerization in the extracellular space. The microfibrils tested in this study were purified via size-exclusion chromatography, which separates structures of high molecular mass (MDa) from those of lower mass (kDa). This means the fractions used should be enriched with only mature fibrillin and collagen VI microfibrils of varying lengths. Theoretically, immature forms of fibrillin-1, already bound to calreticulin and PDI, could have associated with these mature microfibrils. However, the N terminus and C terminus of immature fibrillins are cleaved by furin (at positions 44 and 1732, respectively) (73, 74) only after secretion from the cell (72). In all samples tested, LC-MS/MS failed to detect any peptides corresponding to these cleaved propeptides. This suggests that immature fibrillin-1 was not detected in any of the samples tested. As such, it is possible that the intracellular proteins calreticulin and PDI were released from cells during the extraction process, where they then associated with the mature fibrillin microfibrils. Another explanation for the presence of PDI in the extracellular space is the recent evidence of its secretion from cells via an activation of αvβ3 integrin (75). In either case, the co-purification of calreticulin and PDI supports their proposed roles in microfibril assembly.

MFAP5, -2, and -4 were also identified in microfibril samples from both eye and skin tissues. Gibson *et al.* (76) previously showed in bovine that MFAP5 (also known as MAGP-2) is localized in CB but not in the zonule, in contrast to MFAP2 (also known as MAGP-1), which was found in both. This fits with our identification, because the microfibrils used in this study were extracted from human CB. However, De Maria *et al.* (22) did detect MFAP5 both in high abundance in the human zonule and in lesser abundance in the bovine zonule. It is possible that either the CB was a contaminant within the De Maria *et al.* (22) zonular samples (as in this study, they also detected the basement membrane proteins nidogen and laminin, which can be attributed to the CB epithelium) or that perhaps there exists a genuine disparity between the composition of human and bovine ciliary zonules.

MFAP4 and -5 are both instrumental to the proper formation and organization of elastic fibers (61, 64) by interacting and co-localizing with fibrillin-1, tropoelastin, and the cross-linking enzyme desmosine as well as promoting tropoelastin self-assembly on top of fibrillin microfibrils. MFAP2 (also known as MAGP-1) binds strongly to fibrillin microfibrils (63, 77) and was found to interact directly with both TGF-β and BMP-7 (78). Disrupting this interaction in mice leads to a marked increase in TGF-β signaling attributed to the loss of its sequestration into the fibrillin microfibril network (79). As such, MFAP2 plays a key role in modulating fibrillin-growth factor signaling. Fibrillin-2, a key component of maturing fibrillin microfibrils in developing elastic (80) and non-elastic (81) tissues, was also identified in microfibril preparations from both eye and skin. It is likely that these fibrillin microfibril-associated proteins were identified from both tissues because of the fundamental role they play in fibrillin microfibril assembly and function.

Fibrillin-2 and MFAP2 and -4, which were identified in both eye- and skin-derived microfibril samples, were not detected in cultured HDF-derived microfibril samples. The observation that all three HDF-derived fibrillin microfibril purifications lacked detection of fibrillin-2, MFAP2, and MFAP4 compared with tissue-derived indicates the possibility that these microfibrils may be immature and functionally impaired in 1) forming mature fibrillin microfibrils, 2) forming elastic fibers, and 3) modulating growth factor signaling. The differences seen in the ultrastructure and presence of key associated proteins, observed in cell-derived fibrillin microfibrils compared with tissue-derived, could be due to the limitations of the cell culture model itself. Removing HDFs from their native, homeostatic environments could have contributed directly to the formation of immature and possibly defective fibrillin microfibrils. Many studies have exclusively used cell culture-derived microfibrils to elucidate their functional role in the ECM (63, 82, 83). The differences seen in this study demonstrate a problem with this approach, as functional observations based on cell-derived fibrillin microfibrils may not necessarily reflect those in native tissue.

The differences in protein presence between HDF- and skin-derived fibrillin microfibril samples may have also contributed to the differences observed in bead morphology. MFAP2, for instance, binds to the fibrillin microfibril bead directly (38). As a consequence, it is possible that the redistribution of HDF-derived microfibril bead height compared with skin-derived (Fig. 4*A*, *iv*) may be due to the loss of these associated proteins from the surface.

### Conclusion

Building upon previous evidence (16, 17), this study has found that not only is fibrillin microfibril bead morphology tissue source–dependent, but fibrillin-1 regional proteolytic susceptibility is too. This study is first to show ultrastructural and compositional changes between human fibrillin microfibrils from elastin-rich (skin) and elastin-poor (ciliary body) tissues, which have evolved to play very different architectural roles. Additionally, this study observed that newly synthesized fibrillin microfibrils derived from HDFs had a different bead morphology and periodicity compared with native skin-microfibrils. This indicated that these newly synthesized microfibrils may be structurally immature in comparison with those developmentally formed in tissue or that they may lack the structure-altering damage accumulation seen in microfibrils from aged tissue. Additionally, this study demonstrated that collagen VI microfibrils, derived from HDFs and skin, are relatively invariable in periodicity and in regional elastase susceptibility in comparison with the fibrillin microfibril. Finally, this study found that analyzing the presence of the fibrillin microfibril-associated proteins within skin, eye, and HDF-derived samples provides insight into the role they play in the elastic fiber and the basement membrane. Additionally, it allowed the potential identification of proteins that could be fundamental to fibrillin microfibril biology regardless of their tissue source and the observation that newly synthesized microfibrils from cell culture lacked some of these proteins.

Although the loss and deterioration of the fibrillin microfibril network in response to chronic photoaging has been observed immunohistochemically (5), the effects on fibrillin microfibril ultrastructure, fibrillin-1 protease susceptibility, and associated protein composition have yet to be studied. The techniques and methodology used in this tissue and culture comparison would be well-suited to this goal.

## Experimental procedures

### Study design

**Figure 7.**
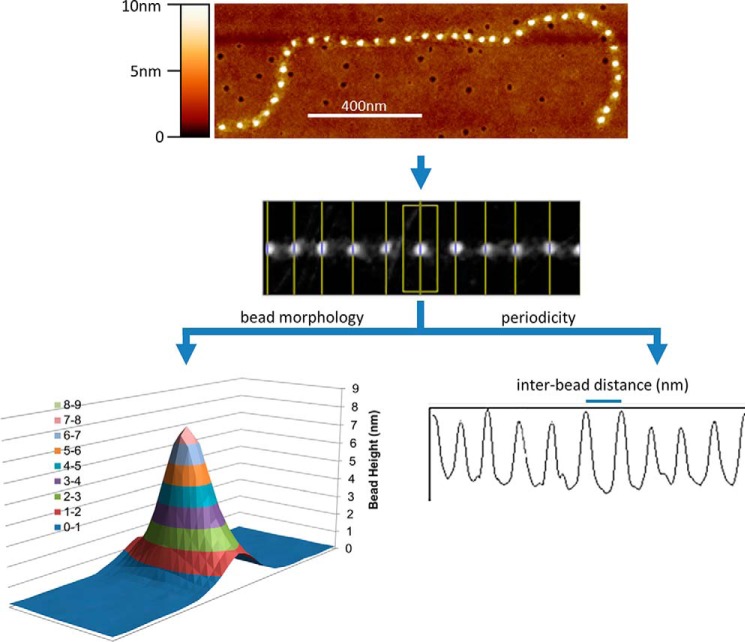
**Ultrastructural measurements of the fibrillin microfibril, performed with AFM.** Fibrillin microfibrils adopt a beads-on-a-string appearance when viewed with AFM. The height maps generated were used to measure and compare the bead morphology and the periodicity (interbead distance) of eye (CB)-, skin-, and HDF-derived fibrillin microfibrils.

Microfibrils were extracted and purified from 1) adult human eye (CB) (*n* = 3; M74 (male aged 74 years), F79, F76), 2) adult human abdominal skin (*n* = 3; F49, F55, F56), and 3) cultured HDFs (*n* = 3; M75, M76, M77). The ultrastructure of these purified fibrillin microfibrils (bead morphology and bead-bead periodicity), from these three different sources, was measured and compared using AFM (Fig. 7). The regional susceptibility of the fibrillin-1 domain structure to elastase digestion was measured and compared by counting the average number of LC-MS/MS–detected peptide spectrum matches (PSMs) from each domain. Finally, the presence of known microfibril-associated proteins was detected and compared for each of these purifications using LC-MS/MS.

Collagen VI microfibrils, which are also present in these purifications, were treated as a control, and their periodicity and COL6A3 regional susceptibility to elastase were compared with those of the fibrillin microfibrils in the same samples.

### Reagents and human tissue and cell acquisition

All chemicals were sourced from Sigma-Aldrich Co. Ltd. (Poole, UK) unless stated otherwise. This study was conducted in accordance with the European Medicines Agency Note for Guidance on Good Clinical Practice and the Declaration of Helsinki (1964) (revised Seoul (2008)). The use of human donor eye tissue was approved by the University of Manchester ethics committee (ethics reference number 11305). Tissue was received within 24 h of corneal dissection (for corneal transplant services) from the Manchester Eye Bank, in accordance with the Human Tissue Act. The CB was carefully dissected from each tissue sample, followed by snap-freezing in liquid nitrogen and storage at −80 °C.

Human abdominal skin samples were acquired from the University of Manchester Skin Health Biobank. This biobank was approved by the North West Research Committee (reference number 09/H1010/10). Samples were snap-frozen in liquid nitrogen and stored at −80^°^C.

Primary human dermal fibroblasts were cultivated from skin biopsies taken from donor photoprotected buttock. The use of this skin was approved by the North West Research Ethics Committee (reference number 14415), where all donors gave written and informed consent. All incubations and cultures were performed at 37 °C (5% CO_2_). Biopsies were incubated in Hanks' balanced salt solution (Fisher Scientific, Loughborough, UK) with 10% dispase overnight. The dermis was then dissected and minced before incubation in fibroblast medium: Dulbecco's modified Eagle's medium (Fisher Scientific) containing 10% fetal calf serum, 1% l-glutamine, 1% amphotericin, and 1% penicillin-streptomycin (Gibco, Paisley, UK). Tissue samples were then cultured with weekly fibroblast medium changes until HDFs could be observed on sample plates.

### Microfibril isolation and purification

Human eye and skin tissue samples were minced and added to a 2-ml aliquot of salt buffer (50 mm Tris-HCl, 400 mm NaCl, and 1 mm CaCl_2_, pH 7.4). 1 mg of bacterial collagenase IA, 0.01 mm phenylmethylsulfonyl fluoride, and 0.03 mm *N*-ethylmaleimide were then added to the tissue, which was digested for 4 h on a rotary mixer, at room temperature (8, 19).

Post-confluent (passage 2) HDFs were maintained for 5 weeks in Dulbecco's modified Eagle's medium + GlutaMAX (Fisher Scientific) containing 10% fetal calf serum and 50 μg/ml 1% penicillin-streptomycin. HDFs were then washed with PBS, and 2 ml of salt buffer was added directly to the culture flasks. 1 mg of bacterial collagenase IA, 0.01 mm phenylmethylsulfonyl fluoride, and 0.03 mm *N*-ethylmaleimide were then added and digested on an orbital shaker for 2 h at room temperature.

Microfibril purification was achieved using an ÄKTA Prime Plus Liquid Chromatography System (GE Healthcare, Little Chalfont, UK). Post-digestion, tissue- and HDF-derived samples were centrifuged at 5000 × *g* for 5 min, and supernatant was run within a column buffer (composed of 50 mm Tris-HCl and 400 mm NaCl at pH 7.4), through a GE HiScale 16/40 column containing Sepharose® Cl2B beads (Sigma-Aldrich), at 0.5 ml/min. Co-purifying fibrillin and collagen VI microfibrils were enriched in the void volume peak, where fractions were collected based on spectrophotometric absorbance at 280 nm (8, 19). Aliquots of the purification were kept for AFM, and the rest were desalted in 0.22-μm–filtered ultrapure water using Slide-A-Lyzer^TM^ MINI dialysis devices (Thermo Fisher Scientific, Paisley, UK) for 4 h at 4 °C. Samples were subsequently frozen at −80^°^C and freeze-dried at −60^°^C for 48 h before storage at −80 °C until their use in MS experiments.

### Microfibril peptide generation using elastase and SMART^TM^ digestion before mass spectrometry

To enhance fibrillin-1 peptide generation, half of the freeze-dried samples were resuspended in 0.1 m Tris-HCl, pH 8.5. Proteins were denatured in 8 m urea, reduced in 10 mm DTT for 30 min at room temperature, and alkylated using 50 mm iodoacetamide for 30 min at room temperature in darkness. The solution was then diluted down to 2 m urea, and elastase (catalogue no. E1250) was added at a 2:1 enzyme/substrate ratio. This was incubated at 37 °C for 4 h. Elastase activity was then quenched with 5% formic acid in ultrapure water.

To enhance microfibril-associated protein peptide generation, the other half of the freeze-dried samples were resuspended in ultrapure water and directly digested for 75 min using a SMART Digest^TM^ kit (Thermo Scientific), which allows the fast digestion of the sample through immobilized trypsin beads, at a high, denaturing temperature (70 °C) (84), as per the manufacturer's instructions. All samples were then desalted using POROS R3 (Life Technologies, Paisley, UK) beads and vacuum-dried before MS analysis.

### Mass spectrometry

All MS was performed by the Biological Mass Spectrometry Core Facility in the Faculty of Biology, Medicine, and Health at the University of Manchester (Manchester, UK). As dictated by their protocols (85, 86), vacuum-dried samples were analyzed by LC-MS/MS using an UltiMate® 3000 Rapid Separation LC (Dionex Corp.; Sunnyvale, CA) and an Orbitrap Elite mass spectrometer (Thermo Fisher Scientific). Peptide mixtures were separated using a gradient from 92% A (0.1% formic acid in water) and 8% B (0.1% formic acid in acetonitrile) to 33% B in 30 min at 300 nl min^−1^, using a 250 mm × 75-μm inner diameter 1.7-μm BEH C18, analytical column (Waters). Peptides were selected for fragmentation automatically by data-dependent analysis.

### Mass spectrometry data analysis

Mass spectra were extracted using extract_msn (Thermo Fisher Scientific) correlated against the Uniprot human database (87) using Mascot version 2.5.1 (Matrix Science, London, UK).

Search parameters were as follows: species, *Homo sapiens*; enzyme, trypsin for SMART^TM^-digested samples and nonspecific for elastase-digested samples; maximum missed cleavages, 1; fixed modifications, carbamidomethyl (mass, 57.02; AA, C); variable modification, oxidation (mass, 15.99; AA, M); peptide tolerance, 10 ppm (monoisotopic); fragment tolerance, 0.6 Da (monoisotopic); searched database, SwissProt_2016_04 (152,544 protein entries).

Data generated were validated using Scaffold (Proteome Software; Portland, OR). Only exclusive, unique peptide counts are reported. False discovery rate (FDR) was calculated by Scaffold using protein and peptide probabilities assigned by the Trans-Proteomic Pipeline and the Protein Prophet^TM^ (88) and Peptide Prophet^TM^ (89) algorithm (Sourceforge, Seattle, WA). Peptide Prophet FDR was thresholded to ≤5%, and Protein Prophet FDR was thresholded to ≤0.1% (minimum of 2 peptides) for every data set.

The mass spectrometry proteomics data have been deposited to the ProteomeXchange Consortium via the PRIDE (90) partner repository with the data set identifier PXD008450 and 10.6019/PXD008450.

### Microfibril atomic force microscopy

Glass coverslips were soaked in absolute ethanol overnight and then attached to metal stubs with clear nail varnish. Samples were pipetted directly onto the coverslips and left for 1 min, so microfibrils could adsorb to the surface. Liquid was removed, and the stubs were left to dry overnight. Stubs were washed three times with ultrapure water and left to dry before being scanned using AFM. Fibrillin and collagen VI microfibrils were imaged using peak force and Scan-Asyst® mode on a Multimode 8 atomic force microscope (Bruker, Billerica, MA), as described previously (18, 19). Using a single, new Scan-Asyst® air tip (Bruker), single fibrillin MFs were captured at 512 pixels/line in 2 × 2-μm scans. This gave a resolution of 3.9 nm/pixel, which was deemed high enough for fibrillin microfibril ultrastructural analysis (18, 19). Fibrillin MFs that were laterally associated with collagen VI microfibrils were omitted from the analysis.

Scans were digitally flattened using the WSxM version 5.0 AFM image processing package (91) and exported in text image format. Height was corrected by subtracting negative background (92). Using ImageJ, fibrillin microfibrils were straightened using the Straighten Curved Objects plugin (93), enabling the generation of 41-pixel-wide images of single straightened fibrillin microfibrils (Fig. 7). LFA image processing software, developed by our group using Microsoft Visual Basic version 6.0 as described previously (94), was then used to specify the location of the maximum height of each bead and create a 15 × 41-pixel snapshot of each individual bead with the height maxima at the central pixel of the image. Maximum bead height and morphology were taken from these snapshots. Fibrillin microfibril periodicity was measured using the Periodicity and Angles software package developed by our group using Microsoft Visual Basic version 6.0 as described previously (51).

## Author contributions

A. E., C. E. G., R. E. B. W., and M. J. S. conceptualization; A. E., R. O., and D. K. data curation; A. E. formal analysis; A. E., C. B., and M. J. S. validation; A. E. investigation; A. E. visualization; A. E., K. T. M., S. M. P., R. O., and D. K. methodology; A. E. writing-original draft; A. E. and M. J. S. project administration; A. E., K. T. M., S. M. P., C. E. G., R. E. B. W., R. O., C. B., D. K., and M. J. S. writing-review and editing; C. E. G., R. E. B. W., and M. J. S. funding acquisition; R. E. B. W. and M. J. S. supervision; R. O. and D. K. resources; A. E. designed, performed all experiments, analyzed all of the data, prepared the figures, and wrote the paper; K. T. M. contributed to cell culture of primary fibroblasts; S. M. P. contributed to the isolation of primary fibroblasts from human skin; C. E. G. and R. E. B. W. contributed to the study design and to the editing of the paper; R. O. and D. K. provided design, technical assistance, and support for all LC-MS/MS; C. B. contributed to the editing of the paper and interpretation of the results; M. J. S. conceived and coordinated the study and contributed to the preparation of the figures and writing of the paper.

## Supplementary Material

Supporting Information

## Footnotes

3 The abbreviations used are:

ECM: extracellular matrix
AFM: atomic force microscopy
TGF: transforming growth factor
BMP: bone morphogenetic protein
CB: ciliary body
MFAP: microfibril-associated protein
HDF: human dermal fibroblast
EMILIN: elastin microfibril interface-located protein
PDI: protein-disulfide isomerase
FDR: false discovery rate
PSM: peptide spectrum match
EGF: epidermal growth factor.
