# Supplementary material for: Structural and compositional diversity of fibrillin microfibrils in human tissues
Source: J Biol Chem. 2018 Feb 16;293(14):5117–33. doi: 10.1074/jbc.RA117.001483 (PMC5892578; doi:10.1074/jbc.RA117.001483)
Supplement: Supporting Information [file supp_293_14_5117__index.html]

Structural and compositional diversity of fibrillin microfibrils in human tissues — Structural diversity in human tissue fibrillin microfibrils — Structural and compositional diversity of fibrillin microfibrils in human tissues — Structural diversity in human tissue fibrillin microfibrils — Supporting Information 

# Structural and compositional diversity of fibrillin microfibrils in human tissues

## Supporting Information

- Description of Supporting Information - Describes and introduces supplemental figure and table
- Figure S1 - The elastase method and advances in MS technology led to the improved detection of COL6A3 peptides compared to previous published efforts. The ability of the elastase method to produce COL6A3 peptides from a single human CB sample (F67) and single human skin sample (F49) is compared to efforts made by Beecher et al. 2011 (55) from a single bovine cornea sample. Beecher et al. identified COL6A3 peptides from Von Willebrand A domains (vWA) N1-N9 of N-terminal globular region, C1 of the C-terminal globular region and from the triple-helix domain (coloured grey since number of peptide hits was unreported). Since the CB is a collagen VI-poor region in comparison to cornea, our methods only detected peptides from vWA domains N1-N4 and C1 of the globular regions from the human CB-microfibril extract (F67) leading to a lower primary coverage than that by Beecher et al. However, when our methods were applied to human skin (F49), a collagen VI-rich tissue, we achieved a COL6A3 primary coverage which was more than double that of Beecher et al. 2011 (55) .
- Table S1 - Combined table of all fibrillin microfibril co-purifying proteins (Protein Prophet FDR&#x2264;0.1%) identified using LC-MS/MS. Peptide spectrum matches (Peptide Prophet FDR&#x2264;5%) are shown for each sample and for each tissue.
