# Supplementary material for: Structural and compositional diversity of fibrillin microfibrils in human tissues
Source: J Biol Chem. 2018 Feb 16;293(14):5117–33. doi: 10.1074/jbc.RA117.001483 (PMC5892578; doi:10.1074/jbc.RA117.001483)
Supplement: Supporting Information [file supp_RA117.001483_134705_2_supp_66963_p3hrc3.pdf]

Structural and compositional diversity of fibrillin microfibrils in human tissues

Alexander Eckersley[1], Kieran T. Mellody[1], Suzanne Pilkington[2], Christopher E.M. Griffiths[2,5], Rachel E.B. Watson[2,5], Ronan O'Cualain[3], Clair Baldock[1,4], David Knight[3], Michael J. Sherratt[1*]

From the [1]Division of Cell Matrix Biology & Regenerative Medicine, [2]Division of Musculoskeletal & Dermatological Sciences, [3]School of Biological Sciences, [4]The Wellcome Trust Centre for Cell-Matrix Research, Faculty of Biology, Medicine and Health, The University of Manchester and [5]NIHR Manchester Biomedical Research Centre, Central Manchester University Hospitals NHS Foundation Trust, Manchester Academic Health Science Centre, Manchester, UK

Supporting Information

Figure S1. The elastase method and advances in MS technology led to the improved detection of COL6A3 peptides compared to previous published efforts. The ability of the elastase method to produce COL6A3 peptides from a single human CB sample (F67) and single human skin sample (F49) is compared to efforts made by Beecher *et al*. 2011 (55) from a single bovine cornea sample. Beecher *et al*. identified COL6A3 peptides from Von Willebrand A domains (vWA) N1-N9 of N-terminal globular region, C1 of the C-terminal globular region and from the triple-helix domain (coloured grey since number of peptide hits was unreported). Since the CB is a collagen VI-poor region in comparison to cornea, our methods only detected peptides from vWA domains N1-N4 and C1 of the globular regions from the human CB-microfibril extract (F67) leading to a lower primary coverage than that by Beecher *et al*. However, when our methods were applied to human skin (F49), a collagen VI-rich tissue, we achieved a COL6A3 primary coverage which was more than double that of Beecher *et al*. 2011 (55) .

Table S1 (uploaded as a separate excel file). Combined table of all fibrillin microfibril co-purifying proteins (Protein Prophet FDR≤0.1%) identified using LC-MS/MS. Peptide spectrum matches (Peptide Prophet FDR≤5%) are shown for each sample and for each tissue.

Fig. S1

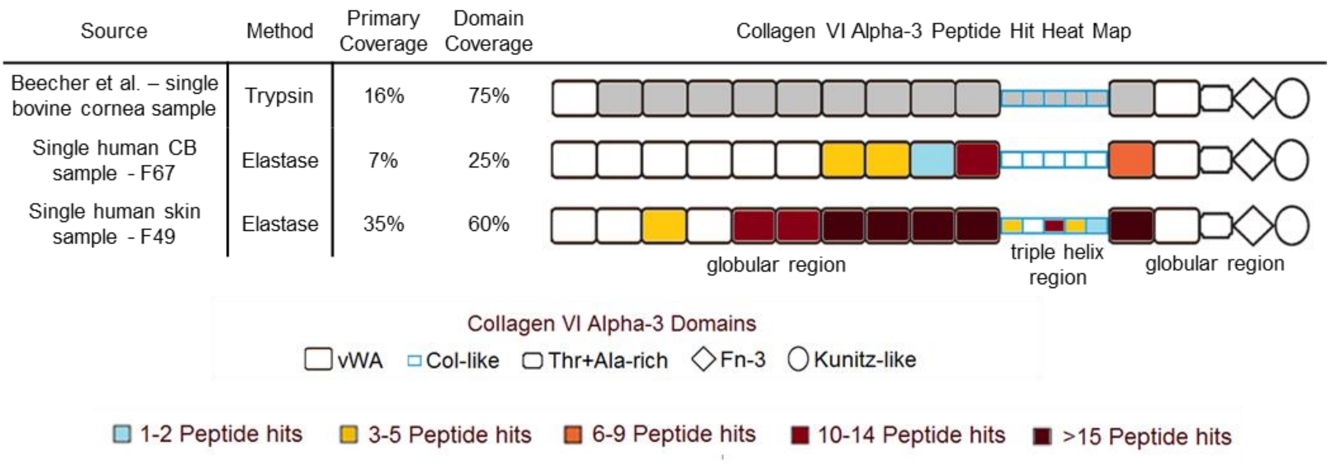
Source
Method
Primary Coverage
Domain Coverage
Collagen VI Alpha-3 Peptide Hit Heat Map
Beecher et al. – single bovine cornea sample
Trypsin
16%
75%
Single human CB sample - F67
Elastase
7%
25%
Single human skin sample - F49
Elastase
35%
60%
globular region
triple helix region
globular region
Collagen VI Alpha-3 Domains
vWA
Col-like
Thr+Ala-rich
Fn-3
Kunitz-like
1-2 Peptide hits
3-5 Peptide hits
6-9 Peptide hits
10-14 Peptide hits
>15 Peptide hits
