# Supplementary figures and images for: Structural and compositional diversity of fibrillin microfibrils in human tissues
Source: J Biol Chem. 2018 Feb 16;293(14):5117–33. doi: 10.1074/jbc.RA117.001483 (PMC5892578; doi:10.1074/jbc.RA117.001483)

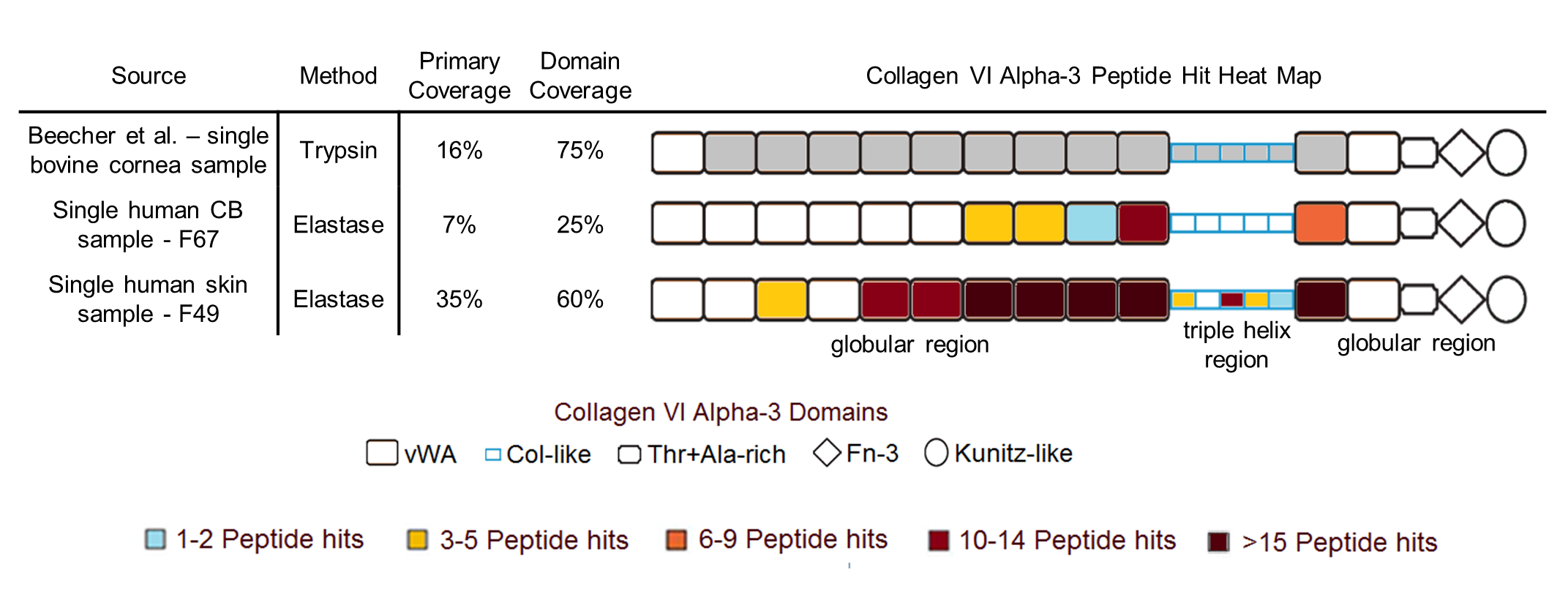

Supplement: Supporting Information [file supp_RA117.001483_134705_2_supp_66969_p3hrc4.tif]
